# Palliative Care Education in Pediatric Cardiology Fellowships: A Survey of Program Directors

**DOI:** 10.1007/s00246-025-03926-1

**Published:** 2025-06-22

**Authors:** Lesje DeRose, Sarah Godfrey, Shabnam Peyvandi, Nicole M. Cresalia, Jill M. Steiner, Emily Morell

**Affiliations:** 1 University of California San Francisco Benioff Children’s Hospitals, San Francisco, CA USA; 2 University of Texas Southwestern Medical Center, Dallas, TX USA; 3 University of Washington Medical Center, Seattle, WA USA

**Keywords:** Congenital heart disease, Palliative care, Fellowship education, Advanced heart disease

## Abstract

With advances in treatment options for severe congenital heart disease, there is a growing population of children and adults living with advanced heart disease, many of whom experience significant long-term comorbidities and uncertain disease trajectories. Although palliative care (PC) plays an integral role in the care of children with advanced heart disease, there is a lack of PC education in pediatric cardiology fellowship training. We distributed a cross-sectional survey to pediatric cardiology fellowship program directors (PDs) nationally (*n* = 58). Survey response rate was 48.3% (28/58). PDs reported PC didactic education in 71.4% (20/28) of programs, with 95% (19/20) reporting this education came from PC specialists, 35% (7/20) from pediatric cardiologists, 25% (5/20) from cardiac intensivists, and 10% (2/20) from general pediatric intensivists. Simulation was used by 10.7% (3/28) of programs. Only one program utilized online modules. Informal bedside teaching occurred in 92.9% (26/28) of programs, and dedicated PC rotations in 25% (7/28). Most programs endorsed annual formal didactics on various PC topics, although some topics were covered more frequently and some never covered by individual programs. Only 53.8% (14/26) of PDs were satisfied with the amount of PC education while 76.9% (20/26) were satisfied with the quality of PC education. Barriers included “too much other content to cover” (77%) and “lack of faculty expertise” (27%). A subset of PDs rated graduating fellows’ skills as less than competent in advance care planning (38.5%), criticall illness communication (15.4%), and complex symptom management (15.4%). With a wide range of PC education practices and high rate of PD dissatisfaction with PC education in their programs, there is a need for standardized PC training recommendations and curriculum.

**Supplementary Information:**

The online version contains supplementary material available at 10.1007/s00246-025-03926-1.

## Introduction

With advancements in treatment options and the associated reduction in mortality for severe congenital heart disease over recent decades, there is a growing population of both children and adults living with advanced heart disease, many of whom experience significant long-term comorbidities and uncertain disease trajectories [1]. Advanced heart disease may result in shortened life expectancy, decreased health-related quality of life, significant symptom burden, increased rates of neurocognitive disorders, and frequent and often unplanned hospitalizations and procedures [2–7]. Palliative care (PC) focuses on improving quality of life, facilitating informed decision-making, maintaining dignity, improving communication, and decreasing distress and symptoms for patients and families with serious or potentially life-limiting illness [8, 9]. This is commonly divided into primary PC, which is performed by the primary team caring for a patient such as their interprofessional cardiology team, and subspecialty PC, which is provided by subspecialty-trained PC providers [10–12].

PC is integral to comprehensive care for children with advanced heart disease [10, 13, 14]. Prior studies on pediatric patients with advanced heart disease have shown a discrepant understanding between physicians and families on disease burden and prognosis, and many pediatric cardiologists report a lack of competence in PC topics, pointing toward a need for improved primary PC skills among pediatric cardiology providers [15, 16]. Despite recent AHA guidelines recommending primary PC training for pediatric cardiologists, no specific guidance currently exist [10]. There is a lack of standardized primary PC education in pediatric cardiology core training and no current understanding of the type or amount of PC training in core pediatric cardiology fellowship programs. Our goal was to assess current PC education and attitudes towards integration of PC into pediatric cardiology fellowship training.

## Methods

We conducted a national cross-sectional survey of categorical pediatric cardiology fellowship program directors (PDs) in the United States. A list of all categorical pediatric cardiology fellowship programs maintained by the Society of Pediatric Cardiology Training Program Directors (SPCTDP) [17] was utilized to identify eligible participants. PDs were emailed directly using individual emails. Initial emails were sent in December 2024. A reminder email was sent via the PD listserv in early January 2025, and final reminder emails were sent in late January 2025. The survey closed in February 2025.

Our survey was modeled after a recent PC education survey performed in adult cardiology [18]. Questions in our survey covered modalities and frequency of PC education as well as PD satisfaction and barriers to PC education (Supplement 1). As there are often misconceptions and differences of opinion on what constitutes palliative care, our survey started with brief definitions of primary and subspecialty palliative care. Questions on frequency and skill rating utilized a 5-point Likert scale [19]. Other questions utilized binary yes/no responses. PDs were also asked to provide information on program location, size, and availability of clinical PC services. All survey questions required responses other than the institution name and an optional-free text response item for additional comments. Survey responses were included in analysis if at least 70% of the survey was completed. The survey was reviewed and approved by the University of California San Francisco Institutional Review Board. Survey response was accepted as informed consent, which was explicitly stated in the invitation to participate. Survey responses were collected using Qualtrics (Provo, UT).

## Results

Of the 64 categorical pediatric cardiology training programs listed in SPCTDP, the personal email addresses of 58 PDs were available for direct contact. The survey was completed by 28 PDs (response rate 48.3%). Program demographics are outlined in Table 1. Every program surveyed had access to inpatient subspecialty PC consultation, 14 (50%) had a pediatric PC fellowship, and 16 (57.1%) had an advanced heart failure/transplant fellowship program.

**Table 1:** Participants’ program and institution characteristics

Characteristics (n = 28)	*n* (%)
Program size (number of fellows)	
1–5	4 (14.3%)
6–10	16 (57.1%)
11–15	3 (10.7%)
16 +	5 (17.9%)
Available services	
Inpatient PC consultation service	28 (100%)
Pediatric PC fellowship	14 (50%)
Advanced heart failure/transplant fellowship	16 (57.1%)

*PC* palliative care

Formal didactics for PC education occurred in 20 (71.4%) programs; these were taught by PC specialists (95%), pediatric cardiologists (35%), pediatric cardiac intensivists (25%), and general pediatric intensivists (10%) (Table 2). For programs with formal PC didactics, all but one reported utilizing PC specialists for this education. Only one program (3.6%) used online or self-paced modules for PC education, endorsing the use of Serious Illness Conversation Training [20] and another unlisted training course. In-person simulation was used by 3 (10.7%) programs, with training provided by pediatric PC specialists in one program, pediatric cardiologists in one program, and pediatric intensivists in one program. Dedicated PC clinical rotations were available in 7 (25%) programs, with one program requiring this rotation. Almost all PDs (92.9%) reported that PC education was provided to their fellows using informal methods such as bedside teaching. This was provided by PC specialists (80.8%), pediatric cardiologists (80.8%), and pediatric intensivists (80.8%) equally.

**Table 2:** Pediatric cardiology fellowship palliative care education delivery

Education Type (*n* = 28)	*n* (%)
Formal didactics (lectures, conferences, journal club)	20 (71.4%)
Taught by (select all that apply):	
PC specialist	19 (95%)
Pediatric cardiologists	7 (35%)
Pediatric cardiac intensivist	5 (25%)
General pediatric intensivist	2 (10%)
Online or self-paced modules	1 (3.6%)
Simulation	3 (10.7%)
Taught by (select all that apply):	
PC specialists	1 (33.3%)
Pediatric cardiologists	1 (33.3%)
Pediatric intensivists	1 (33.3%)
Dedicated PC rotation available	7 (25%)
Informal PC education (bedside teaching, etc.)	26 (92.9%)
Taught by (select all that apply):	
PC specialists	21 (80.8%)
Pediatric cardiologists	21 (80.8%)
Pediatric intensivists	21 (80.8%)

*PC* palliative care

**Fig. 1:**
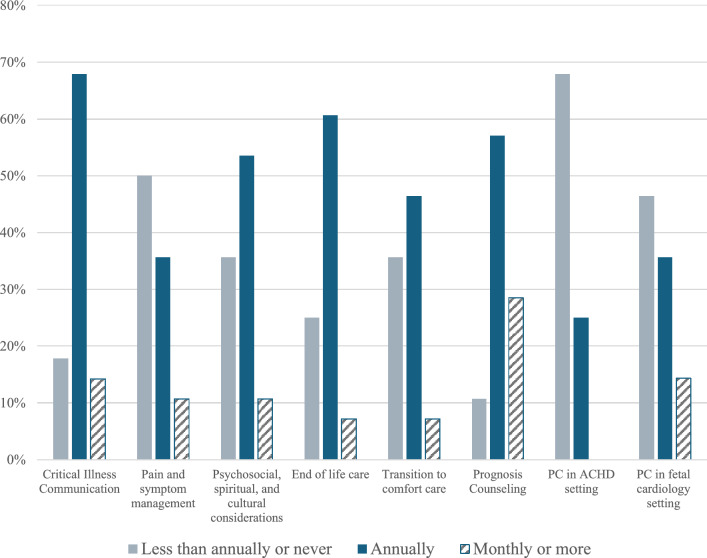
Frequency of PC education by topic (*n* = 28). PD-reported frequency of PC education topics, divided into topics that were covered less than annually or never, annually, or monthly or more than monthly

Most programs endorsed education on a wide range of PC topics annually or more frequently (Fig. 1). However, there were programs that taught some PC topics never or less than annually: critical illness communication in 5 (17.9%) programs, pain and symptom management in 14 (50%) programs, and counseling related to prognosis in 3 (10.7%) programs. More than half of programs (67.9%) taught PC topics related to adult congenital heart disease less than annually, and almost half (46.4%) taught PC in the fetal setting less than annually. Just over half of PDs (53.8%) endorsed satisfaction with the amount of PC education in their program, and 20 (76.9%) endorsed satisfaction with the quality of PC education.

**Fig. 2:**
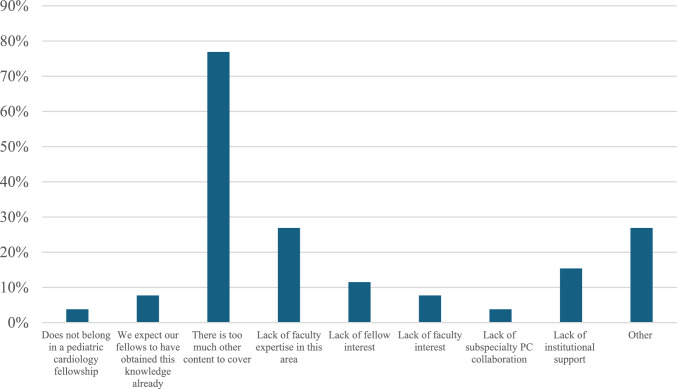
Barriers to providing PC education in pediatric cardiology fellowship (*n* = 26). Barriers selected by PDs to providing PC education during categorical pediatric cardiology fellowship training

Multiple barriers were identified in providing PC education (Fig. 2). The amount of other content to cover was by far the most commonly reported barrier (76.9%), followed by lack of faculty expertise (26.9%). Lack of faculty interest (7.7%), lack of fellow interest (11.5%), and lack of institutional support (15.4%) were also reported. Least commonly selected barriers included not feeling that PC topics belong in pediatric cardiology fellowship training (3.8%) and expecting fellows to have already obtained primary PC training (7.7%). Free text responses highlighted the range of PC educational approaches and lack of standardization across programs.

**Fig. 3:**
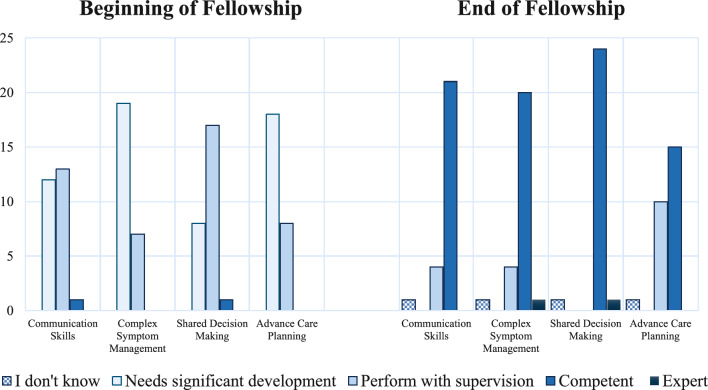
Rating of pediatric cardiology fellows’ PC skills at beginning and end of fellowship. PD rating of fellow’s ability in different primary PC competencies at the beginning (left) and end (right) of categorical pediatric cardiology fellowship training. (*n* = 26)

PDs reported lower PC skills in their fellows at the beginning compared to the end of fellowship (Fig. 3). A subset of PDs rated their average graduating fellow as less than competent in advance care planning (38.5%), communication skills and critical illness conversation (15.4%), and complex symptom management (15.4%). Nearly all PDs felt that fellows graduating from their programs were competent in shared decision making.

## Discussion

Our survey demonstrated a wide range of PC educational practices among categorical pediatric cardiology fellowship programs. While most programs provided some form of PC education to their fellows, the amount and modality varied widely between programs, from very minimal PC exposure to required core PC rotations. Almost half of the participating PDs reported dissatisfaction with the current amount of PC education in their fellowship programs. The majority of PDs highlighted the difficulty of incorporating PC training into already busy schedules and full curricula. Despite the availability of many online and self-paced PC teaching curricula, only one program endorsed the use of this approach. Programs such as VitalTalk (www.vitaltalk.org) and other (both in-person or virtual) modules have been utilized in other training environments with improvement in trainee comfort in PC topics, including CardioTalk for adult cardiology trainees [21–26]. While prior studies have pointed towards the efficacy of simulation in PC training for pediatric providers, only three programs utilized simulation-based PC training [27–29]. Didactic-based curricula in PC topics, such as mental health and ethics, have also been shown to help providers gain confidence in PC domains [30, 31]. One barrier to offering PC eduction may be PC curriculum development. Utilizing curriculum that has already been developed for pediatrics may help lower the barrier to offering PC education, including programs such as EPEC-Pediatrics and VitalTalk, which offer multi-modality training in PC topics [32, 33].

Prior studies have highlighted a lack of confidence in PC skills among pediatric cardiology providers, including the ability to prognosticate and care for children with advanced heart disease, pointing towards a need for further training in these areas [16]. Similar sentiments have been reported in other related training programs, such as adult cardiology, adult congenital heart disease, pediatrics, and intensive care settings [32–36]. Minimal exposure to PC topics in training programs and limited assessment on medical examinations have been identified as barriers to achieving PC competencies [18, 37–39]. In our survey, topics such as transition to comfort care were taught less than annually in more than a third of programs, despite the majority of childhood advanced heart disease deaths occurring as a direct result of discontinuation of disease-directed interventions [40]. Perhaps consideration of more frequent or in-depth training in this topic would be beneficial. Importantly, formal PC training has been shown to improve provider confidence in communication and advance care planning topics, which were both highlighted by PDs as areas that graduating fellows may benefit from more comprehensive training [41]. A better understanding within pediatric cardiology of when subspecialty PC is indicated may also be beneficial, as earlier involvement with PC teams has been linked to a wide range of clinical benefits for patients, including fewer hospitalizations and invasive interventions, decreased anxiety, and improved communication [42]. Interventions trialed in other pediatric subspecialties to reduce barriers to specialty PC consultation have been demonstrated to be feasible and effective [43].

Despite recommendations to include core primary PC competencies in pediatric cardiology training, no specific guidelines exist [10]. In contrast, several papers have proposed essential PC training and competencies in adult cardiology training programs [18, 37]. Tailoring training specifically to topics that a subset of PDs reported notable lack of graduating fellow skill, such as advance care planning, may be a fruitful first step towards greater PC competency. Several effective training modules in advance care planning have been developed and could be recommended and adapted for pediatric cardiology fellows [26, 44, 45]. Importantly, our survey highlighted the lack of national standards as a barrier to PC education, pointing towards a need to create pediatric specific PC training standards.

While our study has a fairly high response rate for a survey-based study, we only received information from approximately half of the surveyed programs. However, responses did come from a wide range of program sizes and geographic locations. Interestingly, there was a dissonance between the high perceived skill level of graduating fellows on PC topics and simultaneous high rate of PD dissatisfaction with PC training during fellowship. As this was a survey of PDs, there is likely a component of over-reporting of PC education and inflation of graduating fellow competency rating due to social desirability bias. Notably, there was no direct assessment of PC competency, and we relied on PD perception of fellows’ skill. Similarly, the ability to assess quality of PC education is limited as much of this education was reported to occur informally and at bedside, without direct PD involvement or observation. However, as PDs are expected to assess graduating fellows in a wide range of pediatric cardiology competencies, we felt that this metric was important to include to see what effect, if any, the lack of standardized PC training had on PD-reported proficiency in PC domains and to help guide educational initiatives. Further research is needed to more accurately assess the proficiency of graduating fellows in PC topics as well as optimal timing, frequency, and format of PC education.

## Conclusion

Our survey was the first to examine PC training practices within categorical pediatric cardiology fellowship programs and found a wide range of approaches to PC education. We highlight the need to standardize PC training recommendations across pediatric cardiology fellowship programs to ensure consistent and adequate PC education.

## Supplementary Information

Below is the link to the electronic supplementary material.

Supplementary file1 (DOCX 17 kb)

## Data Availability

The data that support the findings of this study are not openly available due to reasons of sensitivity and are available from the corresponding author upon reasonable request. Data are located in controlled access data storage in Qualtrics associated with the University of California San Francisco.
