# Supplementary material for: Palliative Care Education in Pediatric Cardiology Fellowships: A Survey of Program Directors
Source: Pediatr Cardiol. 2025 Jun 22;47(4):1513–9. doi: 10.1007/s00246-025-03926-1 (PMC12945887; doi:10.1007/s00246-025-03926-1)
Supplement: Supplementary file 1 — Supplementary file1 (DOCX 17 kb) [file 246_2025_3926_MOESM1_ESM.docx]

**Supplement**

Palliative Care Education Survey

Introduction:

We are interested in better understanding current palliative care educational content in pediatric cardiology fellowship programs.

Palliative care is focused on improving quality of life and relieving distress for patients and families facing serious or life-limiting illness through symptom management, decision making support, guidance around advance care planning, and coordination of care.

Primary palliative care, done by the interprofessional cardiology team, involves the primary team’s role in helping patients and families understand prognosis, process adverse or unexpected events, and address quality of life, symptom management, and decision making. In contrast, secondary or subspecialty palliative care refers to consultation from a subspecialty-trained palliative care team, often related to complex symptoms or decision making, including conflict resolution and transition to end of life care.

We are asking all program directors of pediatric cardiology fellowship programs to complete this survey. Being in this study is optional. If you choose to be in the study, please click next to start our survey. It should take less than 10 minutes to complete. We will keep the data we collect confidential, and we will not share your personal information with anyone outside the research team.

Survey:

1. Please provide the name of your program (free text, optional)
2. What is your role?*
3. Program director
4. Associate program director
5. Other (free text)
6. What is the total number of general cardiology fellows in your program?
   1. 1-5
   2. 6-10
   3. 11-15
   4. 16+
7. In which state is your program located? (drop-down menu-list of states)
8. Which of the following does your institution have? (please select all that apply)
   1. Inpatient palliative care consultation service
   2. Pediatric palliative care fellowship
   3. Advanced heart failure/transplant fellowship
9. Do you provide palliative care education for your fellows using didactics? (lectures, conferences, journal clubs, etc.)
   1. Yes
   2. No

If yes, what type of provider teaches this? (select all that apply)

- - - 1. Pediatric palliative care specialists
      2. Pediatric cardiologists
      3. General pediatric intensivists
      4. Pediatric cardiac intensivists
      5. Other (free text)

1. Do you provide palliative care education for your fellows using ONLINE/SELF-PACED modules?
   1. Yes
   2. No

If yes, please choose all that apply:

- - - 1. Vital Talk
      2. Center to Advance Palliative Care Modules
      3. American College of Cardiology education on website
      4. FAST Facts
      5. EPEC (Education in Palliative and End-of-Life Care)
      6. American Association of Hospice & Palliative Care Education
      7. Serious Illness Conversation Training
      8. Other (Free Text)

1. Do you provide palliative care education using simulation? (in-person)
   1. Yes
   2. No

If yes, who provides this: (please select all that apply)

- - - 1. Pediatric palliative care specialists
      2. Pediatric cardiologists
      3. Pediatric Intensivists
      4. Other (free text)

1. Do you offer a dedicated Palliative Care clinical rotation for your fellows?
   1. Yes
   2. No

If yes, is this a required or elective rotation?

a. Required

b. Elective

1. Do you provide palliative care education using informal methods such as bedside teaching?
   1. Yes
   2. No

If yes, who provides this (please select all that apply):

1. Pediatric palliative care specialists
2. Pediatric cardiologists
3. Pediatric Intensivists
4. Other (free text)
5. How frequently do you have formal didactics on palliative care in your fellowship program on the following content?

0= never, 1 = less than annually, 2 = annually, 3 = monthly, 4= more than monthly, , 5 = don’t know

- 1. Communication (e.g., critical illness conversations, advance care planning, goals of care)
  2. Pain and symptom management
  3. Psychosocial, spiritual and cultural aspects of caring for children with heart disease
  4. End-of-life care
  5. Deactivation or withdrawal of cardiac or life support devices
  6. Counseling related to prognosis (expected lifespan, burden of disease)
  7. Palliative care specifically related to adult congenital heart disease
  8. Palliative care in the fetal cardiology setting

1. Are you satisfied with the current amount of palliative care education in your fellowship program?
2. Yes
3. No
4. Are you satisfied with the current quality of palliative care education in your fellowship program?
5. Yes
6. No
7. What barriers are there to providing palliative care education in your fellowship program? (please select all that apply)
8. This content does not belong in a pediatric cardiology fellowship
9. We expect our fellows to have obtained this knowledge during their residency
10. There is too much other content to cover
11. Lack of faculty expertise in this area
12. Lack of fellow interest
13. Lack of faculty interest
14. Lack of subspecialty palliative care collaboration
15. Lack of institutional support
16. Other (free text)
17. How would you rate your average fellows’ skill level in the following competencies at the BEGINNING of fellowship?

0 –I do not know; 1= Needs significant development, 2 – Able to perform skill with supervision, 3– Competent and ready for independent practice; 4=expert

- 1. Communication skills, critical illness conversations
  2. Complex symptom management
  3. Shared decision making
  4. Advance care planning and goals of care

1. How would you rate your average fellows’ skill level in the following competencies at the END of fellowship?

0 –I do not know; 1= Needs significant development, 2– Able to perform skill with supervision, 3– Competent and ready for independent practice; 4=expert

- 1. Communication skills and critical illness conversations
  2. Complex symptom management
  3. Shared decision making
  4. Advance care planning and goals of care

1. Anything else you would like to share? (for example- barriers or successes) (optional, free text)

*Of note, this survey was only directly distributed to program directors. As the survey was also sent via listserv, we included this question to ensure that we did not include responses from individuals who were not PDs.
